# Evaluation of the health risk using multi-pollutant air quality health index: case study in Tianjin, China

**DOI:** 10.3389/fpubh.2023.1177290

**Published:** 2023-06-07

**Authors:** Yu Wang, Mo Dan, Yan Dou, Ling Guo, Zhizhen Xu, Ding Ding, Mushui Shu

**Affiliations:** ^1^Center of Excellence for Environmental Safety and Biological Effects, Beijing Key Laboratory for Green Catalysis and Separation, Department of Chemistry, Beijing University of Technology, Beijing, China; ^2^Institute of Urban Safety and Environmental Science, Beijing Academy of Science and Technology, Beijing, China; ^3^School of Energy and Environmental Engineering, University of Science and Technology Beijing, Beijing, China

**Keywords:** air quality, air pollution, health risk, disease, air quality health index, air quality index

## Abstract

**Introduction:**

Air pollution imposes a significant burden on public health. Compared with the popular air quality index (AQI), the air quality health index (AQHI) provides a more comprehensive approach to measuring mixtures of air pollutants and is suitable for overall assessments of the short-term health effects of such mixtures.

**Methods:**

We established an AQHI and cumulative risk index (CRI)-AQHI for Tianjin using single–and multi-pollutant models, respectively, as well as environmental, meteorological, and daily mortality data of residents in Tianjin between 2018 and 2020.

**Results and discussion:**

Compared with the AQI, the AQHI and CRI-AQHI established herein correlated more closely with the exposure-response relationships of the total mortality effects on residents. For each increase in the interquartile range of the AQHI, CRI-AQHI and AQI, the total daily mortality rates increased by 2.06, 1.69 and 0.62%, respectively. The AQHI and CRI-AQHI predicted daily mortality rate of residents more effectively than the AQI, and the correlations of AQHI and CRI-AQHI with health were similar. Our AQHI of Tianjin was used to establish specific (S)-AQHIs for different disease groups. The results showed that all measured air pollutants had the greatest impact on the health of persons with chronic respiratory diseases, followed by lung cancer, and cardiovascular and cerebrovascular diseases. The AQHI of Tianjin established in this study was accurate and dependable for assessing short-term health risks of air pollution in Tianjin, and the established S-AQHI can be used to separately assess health risks among different disease groups.

## 1. Introduction

Rapid economic development and urbanization has led to air pollution becoming the greatest environmental threat to global public health and imposing a huge burden of disease on the public. Data published in 2016 by the World Health Organization (WHO) (1) shows that 4.2 million persons die annually from ambient air pollution worldwide, and 92% of the global population live with air quality standards that exceed those recommended by the World Health Organization (WHO) (2), mainly in Asia and Africa (3). Although the Chinese government has implemented several measures to reduce air pollution, control remains a long-term process. According to the recently published State of Ecology and Environment Report of China 2021 (4), the average concentrations of particulate matter (PM_2.5_ and PM_10_), ozone (O_3_), sulfur dioxide (SO_2_), nitrogen dioxide (NO_2_), and carbon monoxide (CO) in China, especially in Beijing-Tianjin-Hebei and surrounding areas were still not optimistic. All these concentrations far exceeded the long-term exposure indexes described in the 2021 Global Air Quality Guidelines published by the WHO (5). These findings showed that persistent air pollution in China is very serious, especially in Beijing-Tianjin-Hebei and surrounding areas. Air pollution adversely affects the health of populations (6–9) and can increase risk of death (10). The burden of disease from air pollution is higher in developing, than in developed countries (11, 12). The public needs to develop awareness of the severity of air pollution and its potential health effects.

The air quality index (AQI) is intended for public use so that individuals are aware of air quality and potential health effects. The AQI uses a simple formula and the data is presented using the well-known ‘traffic light’ scale, making it straightforward for the public to comprehend (13). However, the AQI reflects air quality only as the amount of a pollutant with the highest subindex without considering the possible combined effects of simultaneous exposure to multiple pollutants and ignoring variations in the characteristics of relationships between health outcomes and air pollutants in different countries or regions (13). Therefore, the index does not reflect the non-threshold concentration response relationship between air pollutants and health risks (14–16). To address the shortcomings of the AQI, the concept of an air quality health index (AQHI) was first proposed in Canada to assess the effects of multiple air pollutants on health and to assess air quality (17). The AQHI has subsequently been investigated in Shanghai (18, 19), Guangzhou (15), Tianjin (20), and Hong Kong (21), China. Du et al. (22) analyzed exposure-response relationships between multiple air pollutants and daily mortality rates in 272 representative cities in China, and established a national AQHI based on PM_2.5_, O_3_, and NO_2_. Others have suggested that because the AQHI is calculated by directly adding excess risk using single pollutant models, the impact of pollutants might be overestimated (23, 24). This issue has been addressed by introducing the cumulative risk index (CRI) to establish the CRI-AQHI (25, 26). The AQHI seems to correlate more closely with health outcomes than the AQI, and provides a more comprehensive method of measuring mixtures of several air pollutants, allowing for a holistic assessment of their effects on health (27). Accumulated time-series and case-crossover studies have provided strong evidence of the acute health effects of air pollution (28, 29). In addition, individuals in populations respond differently, even when exposed to the same amount of air pollution. Exploring the effects of air pollution on the health of populations with various diseases is important to develop an in-depth under-standing of the mechanisms of air pollution health hazards and strategies for population-based prevention.

Air quality is affected by factors including seasons, time, geographic locations and weather changes. Therefore, identifying ambient air pollution levels, and effectively communicating the significance of air pollution to public health are hot spots for epidemiological research. The relationship between air pollution and health risk varies greatly among regions and times. Thus local AQHIs should be established instead of directly applying the results of other cities or earlier AQHI studies. This would help to cope more effectively with the regional complexity and variability of air pollutants and to ensure that AQHI results faithfully reflect the impact of local air quality on public health.

## 2. Methodology

### 2.1. Study area

Air pollution in the Beijing-Tianjin-Hebei region has become an extremely serious problem. The rapid development of the Chinese economy has led to this region becoming the capital economic circle of China. It has a strong industrial base and a developed commodity economy, and Tianjin is the most important industrial city in the region. It is representative of cities where the air is so polluted that it confers great risk on the health of the population. An AQHI has been established for Tianjin using principal component analysis (PCA) of data from 2014 to 2017 (20). However, it did not consider varied levels of susceptibility of different populations toward air pollution hazards and a stratified analysis of various disease groups was not included. Tianjin formulated an action plan (2018–2020) for ecological environmental protections to accelerate improvements in the quality of local ambient air (30). Therefore, a locally applicable exposure-response relationship model is needed based on recent health data and air pollution data in Tianjin to reconstruct its AQHI. The effects of air pollution on the health of populations with various diseases should be analyzed to understand the health risks of air pollution.

### 2.2. Data sources

Average daily concentrations of air pollutants (PM_2.5_, PM_10_, O_3_, SO_2_, NO_2_, and CO) at various monitoring sites in Tianjin during 2018–2020 were obtained from the China National Environmental Monitoring Station. The daily air pollutant concentrations were calculated by taking the average of the data collected from the 15 monitoring stations in Tianjin. Daily meteorological data for Tianjin were obtained from the National Meteorological Observing Station in Tianjin Urban district, including daily maximum, minimum, and average temperatures (AVET; °C) and daily average relative humidity (AVEH; %). Daily mortality data for Tianjin residents during the study period were obtained from the Chinese Center For Disease Control And Prevention (CCDC) Cause of Death Reporting System, including sex, age, location, and underlying cause of death. The International Classification of Diseases (ICD) codes were A00-R99, and S00-Z99 were excluded. The ICD codes of cardiovascular diseases, lung cancer, and chronic respiratory diseases were I00-I99, C33-C34, and J30-J98, respectively. AQI data were obtained from the website.^1^

The data were then integrated, anomalies were removed. For the pollutant data, the missing data were identified and generated via linear interpolation. The number of deaths resulting from Covid-19 was deleted from non-accidental death data. Considering that PM_2.5_ is more hazardous to humans, we selected PM_2.5_ as the representative particulate matter.

### 2.3. Statistical analysis

We used Spearman correlations method for the descriptive analysis. We used the Generalized Additive Model (GAM), which is a time series analysis, to estimate the exposure-response relationship between air pollutant concentrations and daily mortality rates in Tianjin. By fitting air pollutants and confounders of unknown forms through parametric and non-parametric methods, respectively, the GAM controlled confounding factors of non-linear relationships associated with death caused by temperature and humidity through smoothing functions. We introduced the control variables of date, temperature, humidity and day of the week as confounders in the GAM. We established an AQHI for Tianjin using single- and multi-pollutant models, respectively, and health and air pollution data from Tianjin between 2018 and 2020. We then used our AQHI for Tianjin to analyze populations with different diseases.

#### 2.3.1. Single-pollutant model

##### 2.3.1.1. Exposure-response relationship

The dependent variable in the single-pollutant model was the daily mortality rate of the population, which follows a Poisson distribution relative to the entire population or time period. Thus, the quasi-Poisson distribution is linked to the GAM to solve the problem of overdispersion of the mortality rate. The following base function was established by Eq. 1 (16):


(1) $$\log E\left(Y_{t}\right)=\beta Z_{t}+ns\left(time,\nu \right)+DOW+ns\left(X_{t},\nu \right)+\alpha$$

where *E(Y_t_)* is the predicted daily mortality of the population on observation day *t*, *Z_t_* is the level (μg/m^3^) of a pollutant on observation day *t*, *β* is the exposure-response relationship coefficient, namely, the daily change in mortality caused by each unit increase in the pollutant, and time is the date variable, which can effectively control long-term and seasonal fluctuations in pollution-mortality series data by choosing an appropriate degree of freedom (*ν*) for a specific date. Sensitivity was analyzed using the Qian etc. (31) and Welty and Zeger (32) approaches, with the annual degree of freedom identified as 8. *DOW* is a dummy variable, namely the effect of the day of the week, which eliminates the impact of short-term natural fluctuations in mortality; *ns* is the natural smoothing spline function, and *ν* is the degree of freedom. It deals with non-linear trends and serial correlations of daily mortality counts along a time axis. That is, confounding factors related to long-term variability over time including long-term trends and meteorological factors are controlled. *X_t_* represents meteorological factors on day *t*, including daily average temperature (°C) and relative humidity (%), with the degrees of freedom of the smoothing function set to 3 during the study (31, 32).

Considering the lagged effect of air pollutants on health, we further examined the effect of air pollutants with different lag structures of single-day lag (distributed lag from Lag0 to Lag7).

Exposure-response relationships were established for each disease (cardiovascular diseases, lung cancer, and chronic respiratory disorders). We analyzed health risk for populations with different types of diseases based on daily mortality data of residents. The effects of each pollutant on the total daily mortality rate of populations with different diseases was calculated through GAM, that is, the increase in the total daily mortality rate of populations with different types of diseases caused by 10 μg/m^3^ increments in pollutant concentrations.

We further investigated the effects of each air pollutant on the health of different populations. We initially calculated their contributions to the increased daily mortality of different populations based on the relationships between the effects of air pollutants on the total daily mortality of populations with different types of diseases in Tianjin and the daily air quality data between 2018 and 2020.

##### 2.3.1.2. Excess rate

With zero air pollutants as the base point, the exposure-response relationship model was used to derive the health exposure-response coefficients of major air pollutants and calculate excess mortality rates caused by daily levels of each pollutant during the studied period using the Eq. 2:


(2) $$ER_{kt}=100\times \left[\left(e^{\beta \times p_{kt}}\right)-1\right]$$

where *ER_kt_* is excess mortality caused by pollutant *k* on day *t*, *β* is the coefficient of the exposure-response relationship estimated by regression models (incremental daily mortality caused by each unit increase in a pollutant), and *P_kt_* is the average concentration of pollutant *k* on day *t*.

The Tianjin air quality health index was established based on excess mortality caused by an increase in the concentration of a unit pollutant calculated by Eq. 3:


(3) $$AQHI=10\times \left(\underset{k=1\ldots n}{\sum }ER_{kt}\right)/\max_{k=1\ldots n}ER_{kt}$$

#### 2.3.2. Multi pollutant model

##### 2.3.2.1. Cumulative risk index

The CRI, originally proposed by Lippmann etc. (33), represents the relative risk of a 1-unit increase in all pollutants versus no increase in any pollutant. Based on the assumption of an additive effect of combined pollutant exposure on mortality, the CRI was calculated by applying a multi-pollutant generalized additive model (GAM) with Eqs. 4, 5:


(4) $$\log E\left(Y_{t}\right)=\overset{p}{\underset{p=1}{\sum }}\beta_{p}Z_{pt}+ns\left(time,\nu \right)+DOW+ns\left(X_{t},\nu \right)+\alpha$$

(5) $$CRI_{t}=\exp\left[\overset{p}{\underset{p=1}{\sum }}\beta_{p}Z_{pt}\right]$$

where *CRI_t_* is the cumulative risk of multiple pollutants on day *t*.

##### 2.3.2.2. Excess rate

The rate of excess mortality caused by daily air pollutant mixtures during the study period was then calculated by Eq. 6:


(6) $$ER_{t}=100\times \left(CRI_{t}-1\right)$$

where *ER_t_* is the excess mortality rate caused by the air pollution mixture on day *t*. The CRI-AQHI is subsequently calculated in the same way as the standard AQHI.

#### 2.3.3. Air quality health index construction

Since the AQHI is a composite indicator of multiple air pollutants, selecting the appropriate pollutant as the primary indicator to establish the AQHI is critical. Which pollutants should be included when establishing AQHIs for different regions has not reached consensus. Early studies mostly used two to three pollutants to evaluate health effects (31, 34–36). In Canada (16) and China (22), AQHIs were established based on PM_2.5_, NO_2_, and O_3_. The AQHI for Shanghai (18) included PM_10_ or PM_2.5_ and NO_2,_ whereas that for Guangzhou (15) included PM_2.5_, SO_2_, NO_2_, and O_3_. The main reason for this is that the composition of multiple air pollutants and population susceptibility differs among regions (15).

In order to evaluate the exposure-response relationships between multiple air pollutants and health risks, we constructed an AQHI for Tianjin by using two types of methods: the standard single pollutant model and a multi-pollutant model that includes a cumulative risk index (CRI).

We selected the regression coefficient β and the pollutant concentration on the best lag days to construct the AQHI for Tianjin, the construction results were as follows Eq. 7:


(7) $$\begin{array}{l} \begin{array}{l} AQHI=10/28.08\times 100\times \left[\exp\left(0.0002036\times PM_{2.5}\right)\right.-1\\ +\exp\left(0.0004737\times NO_{2}\right)-1 \end{array}\\ \begin{array}{l} +\exp\left(0.0004444\times O_{3}\right)-1\\ +\exp\left(0.0021855\times SO_{2}\right)-\left.1\right] \end{array} \end{array}$$

Referring to the single-pollutant model, we selected the best lag days for each air pollutant, and the multi-pollutant GAM was applied to construct the CRI-AQHI of Tianjin, the construction results were as follows Eq. 8:


(8) $$\begin{array}{l} CRI-AQHI=10/21.61\times \exp\left(\begin{array}{l} 0.00004821\times PM_{2.5}+\\ 0.0002077\times NO_{2}+\\ 0.0004542\times O_{3}+\\ 0.001689\times SO_{2} \end{array}\right) \end{array}$$

To further investigate the effects of air pollutant on the health of different disease groups, we constructed the S-AQHI for different disease groups in Tianjin, the construction results were as follows Eqs. 9–11:

Lung cancer


(9) $$\begin{array}{l} \begin{array}{l} S-AQHI_{LC}=10/41.52\times 100\times \left[\exp\left(0.0002278\times PM_{2.5}\right)\right.-1+\\ \exp\left(0.0008862\times NO_{2}\right)-1 \end{array}\\ \begin{array}{l} \;\;+\exp\left(0.0004592\times O_{3}\right)-1+\\ \exp\left(0.0042926\times SO_{2}\right)-\left.1\right] \end{array} \end{array}$$

Cardiovascular disease


(10) $$\begin{array}{l} \begin{array}{l} S-AQHI_{CCVD}=10/29.22\times 100\times \left[\exp\left(\begin{array}{l} 0.0002110\times \\ PM_{2.5} \end{array}\right)\right.-1+\\ \exp\left(\begin{array}{l} 0.0005296\times \\ NO_{2} \end{array}\right)-1 \end{array}\\ \begin{array}{l} \;\;+\exp\left(0.0004846\times O_{3}\right)-1+\\ \exp\left(0.0019145\times SO_{2}\right)-\left.1\right] \end{array} \end{array}$$

Chronic respiratory disease.


(11) $$\begin{array}{l} \begin{array}{l} S-AQHI_{CRD}=10/62.08\times 100\times \left[\exp\left(\begin{array}{l} 0.0004801\\ \times PM_{2.5} \end{array}\right)\right.-1+\\ \exp\left(0.0022179\times NO_{2}\right)-1 \end{array}\\ \begin{array}{l} \;\;+\exp\left(0.0005658\times O_{3}\right)-1+\\ \exp\left(0.0056892\times SO_{2}\right)-\left.1\right] \end{array} \end{array}$$

### 2.4. Validity analysis of the AQHI

The daily AQI and AQHI for the study period were descriptively analyzed using the indicators, mean, standard deviation, minimum, maximum, lower quartile (P25), median (P50), upper quartile (P75) and IQR. The differences between the two indices were then compared. The daily values of the AQI and AQHI during the study period were incorporated into separate time series models and the relative degree of daily variation in the AQI and AQHI differed. Thus, we used IQR as a scale to determine the ability of the AQI and AQHI to predict health hazards and to assess the validity of the AQHI.

## 3. Results

### 3.1. Health effects of air pollutants in Tianjin

**Figure 1:**
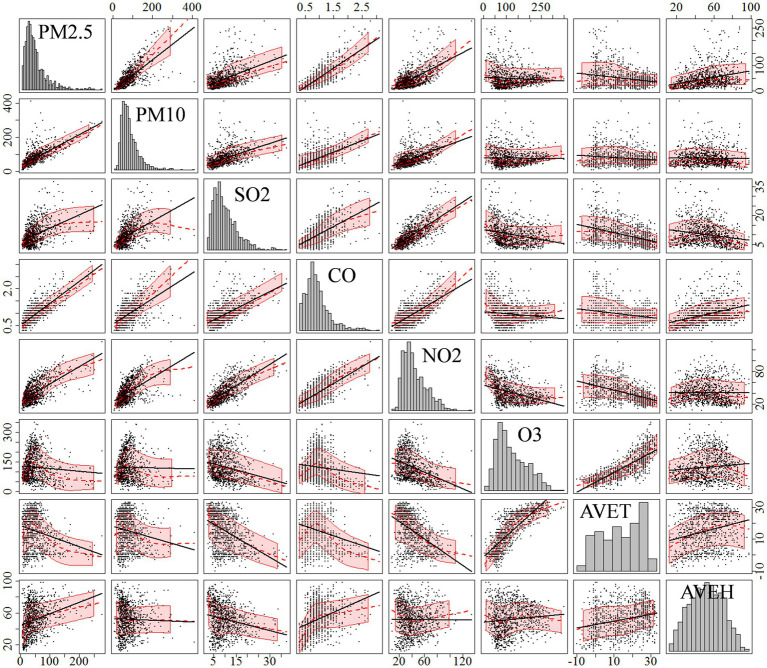
Correlation matrix analysis of environmental variables in Beijing from 2018 to 2020.

Supplementary Table S1 shows the environmental data collected at national monitoring stations, as well as meteorological, and health data for Tianjin for the period 2018–2020. The environmental data showed that the annual average concentrations of PM_10_, PM_2.5_, O_3_, and NO_2,_ were 78.74 μg/m^3^, 50.31 μg/m^3^, 123.74 μg/m^3^and 41.95 μg/m^3^, respectively, all of which far exceeded the long-term exposure targets proposed in the 2021 edition of the WHO Global Air Quality Guidelines (5). In terms of domestic standards, the annual average concentrations of PM_10_, PM_2.5_, and NO_2,_ exceeded the Grade 2 national standards for annual average concentrations (70 μg/m^3^, 35 μg/m^3^, and 40 μg/m^3^). Figure 1 shows correlations between air pollutants and meteorological factors in Tianjin. PM_10_, PM_2.5_, SO_2_, CO, and NO_2_ significantly and positively correlated (*p* < 0.05). O_3_ was significantly positively correlated with daily average temperature, while PM_10_, PM_2.5_, CO, SO_2_, and NO_2_ were all negatively correlated with daily average temperature. PM_2.5_, CO, and O_3_ were all positively correlated with daily average humidity.

**Figure 2:**
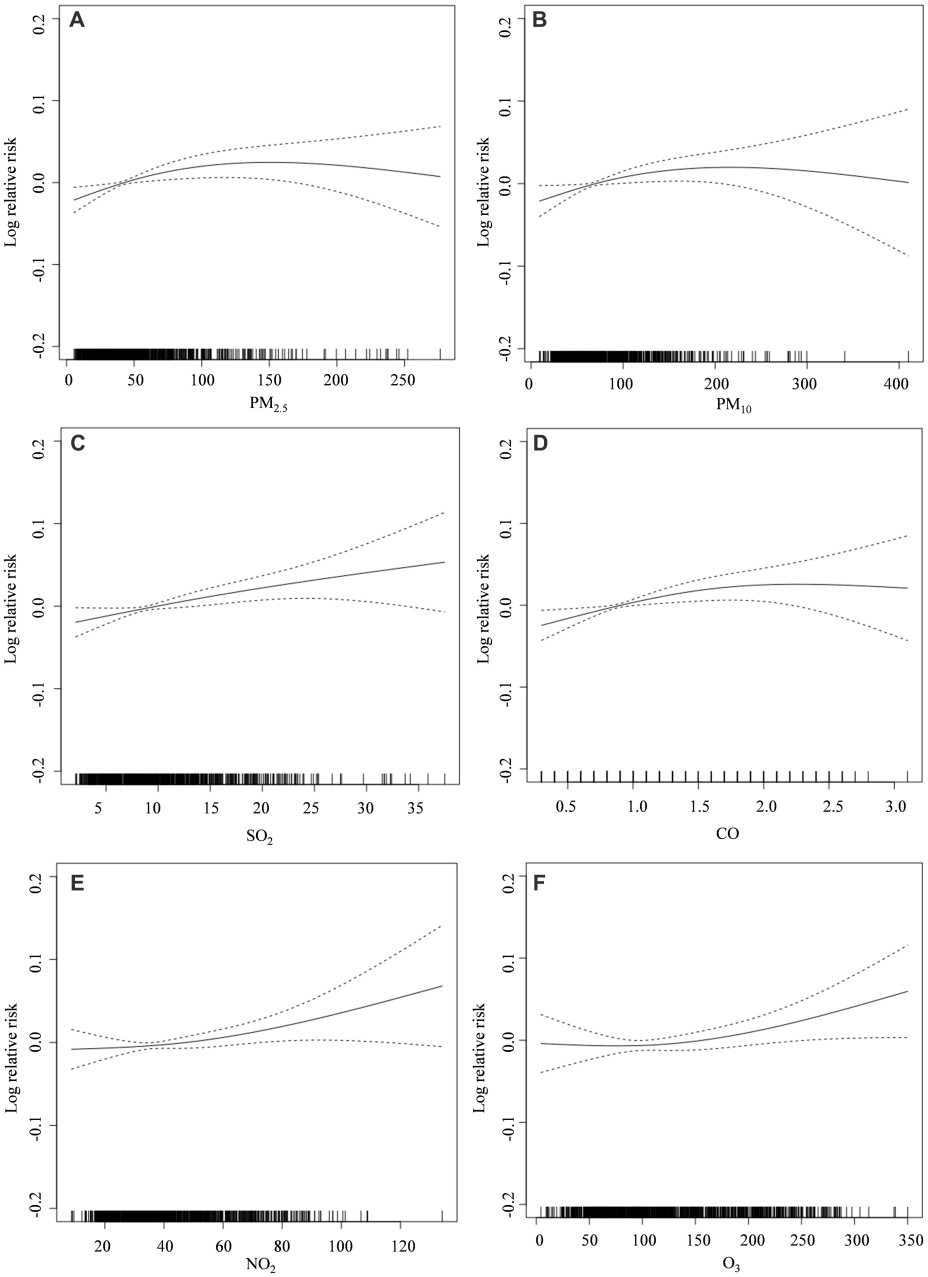
Exposure–response relationship graph of air pollutant concentrations and non-accidental mortality in Tianjin (2018–2020): **(A)** PM_2.5_; **(B)** PM_10_; **(C)** SO_2_; **(D)** CO; **(E)** NO_2_; **(F)** O_3_. The solid line represents the log relative risk of mortality, and the dashed lines represent the 95% confidence interval of the log relative risk.

Figure 2 shows that the exposure-response relationship between SO_2_ and non-accidental mortality rate is largely linear; CO, NO_2_ and O_3_ positively correlated with the non-accidental mortality rate; PM_10_ and PM_2.5_ have largely monotonically increasing exposure-response relationships with non-accidental mortality rates in the P75 concentration range (96.9 μg/m^3^ and 61.3 μg/m^3^), indicating that PM_10_ and PM_2.5_ positively correlated with the non-accidental mortality rate. The overall fluctuation of the CO concentration was small with little effect, and was not selected. We finally established the AQHI based on the air pollutants, PM_2.5_, NO_2_, SO_2_, and O_3_.

### 3.2. Construction results analysis of the Tianjin AQHI

**Figure 3:**
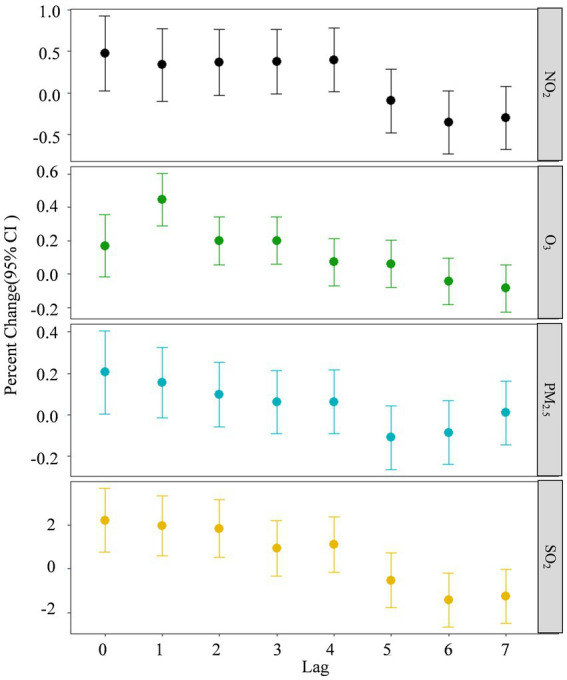
Exposure-response relationships between four air pollutants and daily mortality rates in Tianjin 2018–2020. Lag 0 is the current day, and lags 1–7 represent lags of 1–7 days, respectively.

Figure 3 shows the results of the exposure-response relationship between each air pollutant and daily mortality in Tianjin between 2018 and 2020. The position and length of the line segments in the figure indicate changes (%) in total daily mortality rates of the population caused by 10 μg/m^3^ increments in the concentration of each air pollutant and the 95% CI. The figure shows that the maximum lagged effect of PM_2.5_, NO_2,_ and SO_2,_ occurs on the same day (lag 0), and that the maximum lagged effect of O_3_ is lag 1.

Supplementary Figure S1 shows the daily trends of each air pollutant, mortality AQI and the AQHI of Tianjin for 2018–2020. The figure shows clear seasonal variations in PM_2.5_, SO_2_, NO_2_, and O_3_, whereas the trends of AQHI and CRI-AQHI were not the same as those of the air pollutant concentrations.

The AQHI of Tianjin established herein used daily mortality data of residents and was thus consistent with the AQHI of Canada, where the AQHI featured a significant right-skewed, rather than normal distribution, and the frequency of AQHI greater than or equal to 7 was less than 10%. Therefore, referring to the Canadian grading standard, the AQHI classifies air quality into levels from 1 to 10, with higher values associated with increased health risks (Table 1).

**Table 1:** AQHI classification standards.

AQHI	Health risk level	Warning colors	Susceptible population	General population
0–3	Low	Green	Normal outdoor activities	Suitable for outdoor activities
4–6	Moderate	Yellow	Susceptible individuals should reduce outdoor activities	No need to reduce daily outdoor activities
7–10	High	Red	Older adults, children and susceptible individuals should reduce outdoor activities	Reduce outdoor activities if you have symptoms such as cough or throat irritation
> 10	Severe	Brown	Older adults, children and susceptible individuals should avoid outdoor activities	Outdoor activity should be reduced for all groups

AQHI, air quality health index.

### 3.3. Validity analysis of the AQHI

We validated the effectiveness of AQHI and CRI-AQHI by comparing them with the AQI. Table 2 shows the statistical results of the daily AQI, AQHI and CRI-AQHI for Tianjin between 2018 and 2020. The P50 values for AQI, AQHI, and CRI-AQHI were 72, 3.76, and 3.72, respectively, and their IQRs were 36, 1.85, and 2.13, respectively.

**Table 2:** Comparative statistics of the AQI and AQHI in Tianjin from 2018 to 2020.

Index	Mean	SD	Min	P25	P50	P75	Max	IQR
AQI	80.13	40.25	21	56	72	92	290	36
AQHI	3.91	1.25	1.49	2.97	3.76	4.82	10	1.85
CRI-AQHI	4.06	1.51	1.53	2.90	3.72	5.03	10	2.13

AQI, air quality index; CRI, cumulative risk index; SD, standard deviation; Min, minimum; P25, lower quartile; P50, median; P75, upper quartile; Max, maximum; IQR, interquartile range.

**Figure 4:**
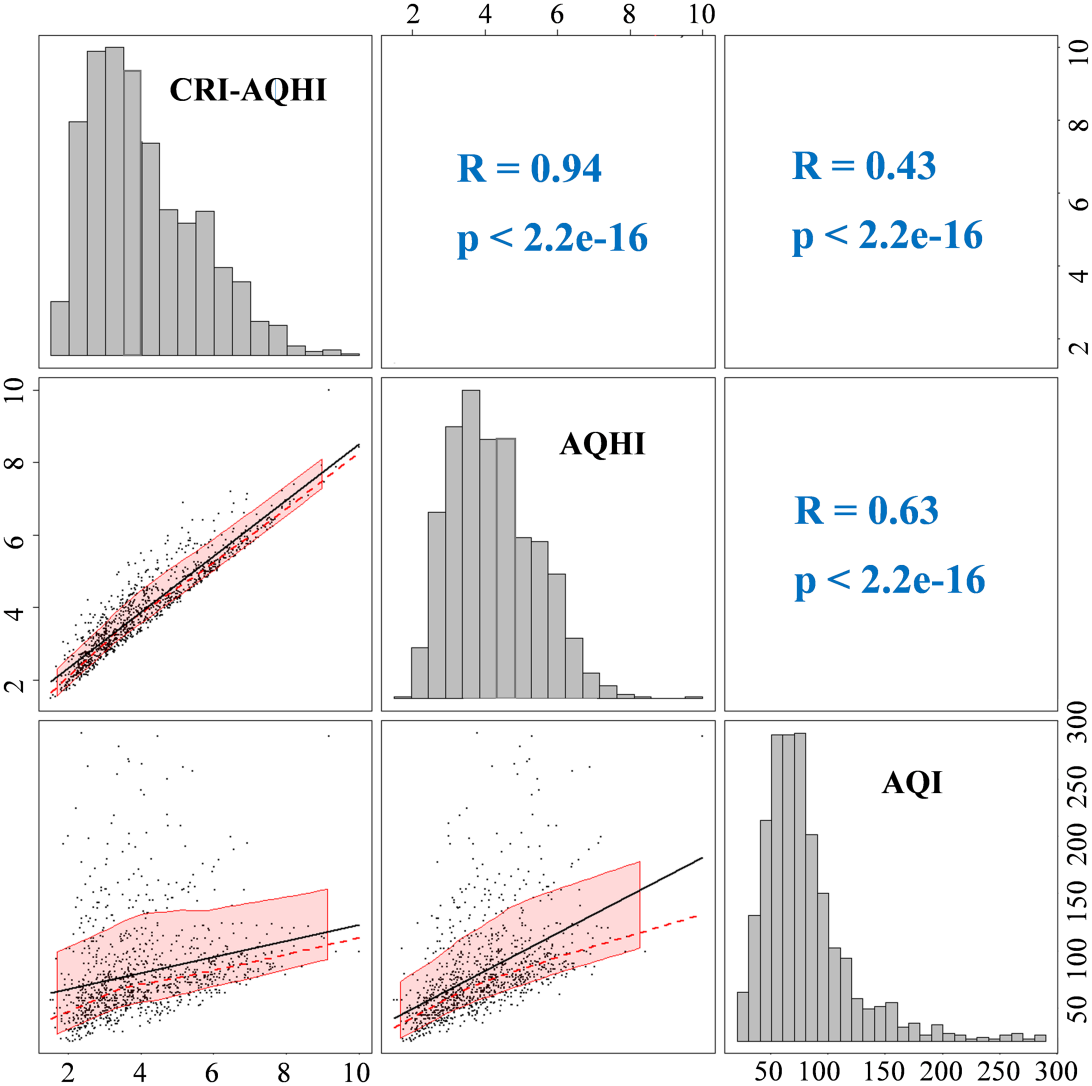
Correlation analysis of AQHI and AQI.

**Figure 5:**
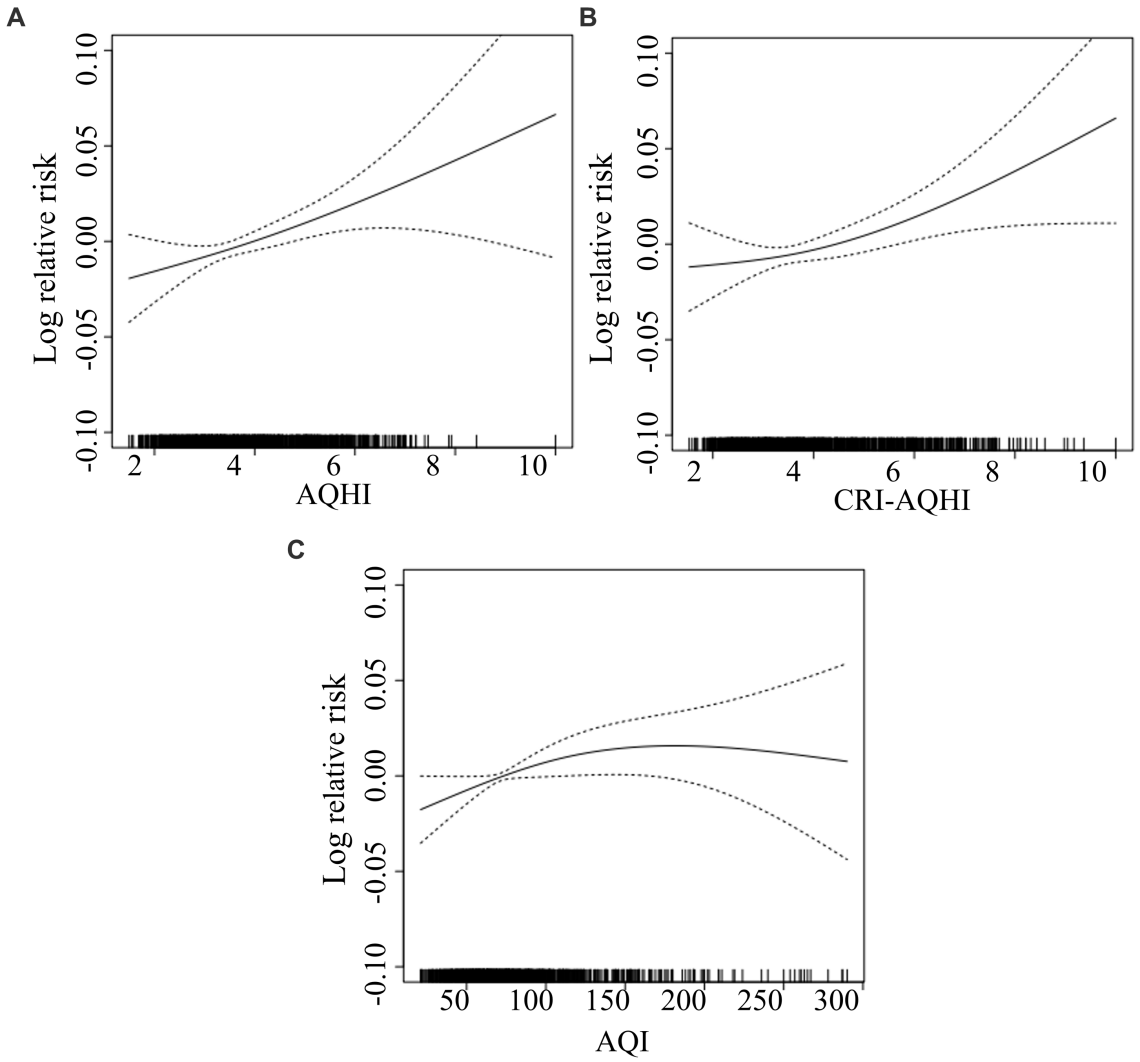
Exposure-response relationship curves of effects of AQHI **(A)** CRI-AQHI **(B)**, and AQI **(C)** on total population mortality.

Figure 4 shows correlations between AQHI and AQI. The Spearman correlation coefficients of AQHI and CRI-AQHI with AQI were 0.63 and 0.43, respectively (both *p* < 0.01), which confirmed the correlation and thus the applicability of AQHI for air quality and health impact assessment. The Spearman correlation coefficient of AQHI and CRI-AQHI was 0.94 (*p* < 0.01), which was significant. Figure 5 shows the results of an analysis of the exposure-response relationship between the AQHI and AQI with total mortality rate for the day. The exposure-response relationship between increasing AQHI and CRI-AQHI indices and increased risk of total resident mortality was essentially linear.

**Figure 6:**
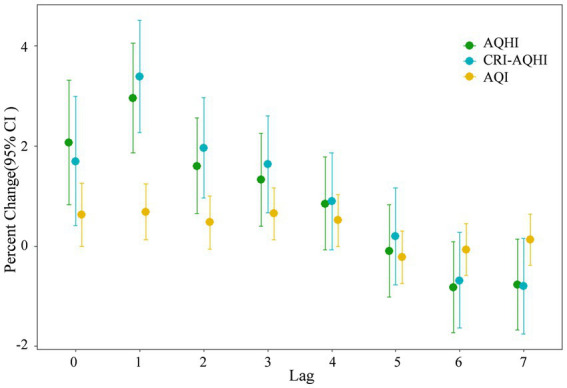
Comparison of correlations between daily mortality and each of AQHI and AQI in Tianjin 2018–2020.

The daily values of AQHI, CRI-AQHI and AQI between 2018 and 2020 were separately incorporated into the time series model. Due to different levels of daily relative changes in AQHI, CRI-AQHI and AQI, we measured their ability to predict health hazards based on IQRs. Figure 6 shows that each increase in the IQR of the AQHI and CRI-AQHI values caused an increase in the total mortality rate of 2.06 and 1.69% on a specific day in Tianjin and correspondingly, each increase of IQR in the AQI resulted in a much lower excess mortality rate of 0.62%. Furthermore, correlations between each of AQHI and CRI-AQHI and health did not significantly differ. Therefore, we selected the coefficients of the single-pollutant model when establishing the AQHI of Tianjin to maintain comparability with previous AQHI findings.

### 3.4. Application of AQHI to populations with different types of diseases in Tianjin

#### 3.4.1. Analysis of exposure-response relationship in populations with different types of diseases

We investigated the relationship of the best lagged data of air pollutants in Tianjin with the total daily mortality rate of populations with different types of diseases based on the results of a time series analysis (Table 3).

**Table 3:** Effects of air pollutants on the total daily mortality rate of groups with different types of diseases in Tianjin (%).

Population classification	PM_2.5_	SO_2_	NO_2_	O_3_
Overall	0.204 (0.004,0.404)	2.210 (0.778,3.662)	0.475 (0.028,0.924)	0.445 (0.289,0.602)
Cardiovascular diseases	0.211 (−0.004,0.427)	1.933 (0.291,3.601)	0.531 (−0.038,1.103)	0.486 (0.283,0.689)
Lung cancer	0.228 (−0.301,0.760)	4.386 (0.571,8.346)	0.890 (−0.280,2.074)	0.460 (0.062,0.860)
Chronic respiratory disease	0.481 (−0.139,1.105)	5.854 (0.827,11.132)	2.243 (0.681,3.829)	0.567 (−0.039,1.177)

Table 3 shows that the effects of PM_2.5_, SO_2_, NO_2_, and O_3_ on total daily mortality differed among the types of diseases. The effects of PM_2.5_, SO_2_, NO_2_, and O_3_ on populations with chronic respiratory diseases and lung cancer were stronger than those for the entire population, and the effects of PM_2.5_, NO_2_, and O_3_ on those with cardiovascular and cerebrovascular diseases were stronger than those for the entire population.

**Figure 7:**
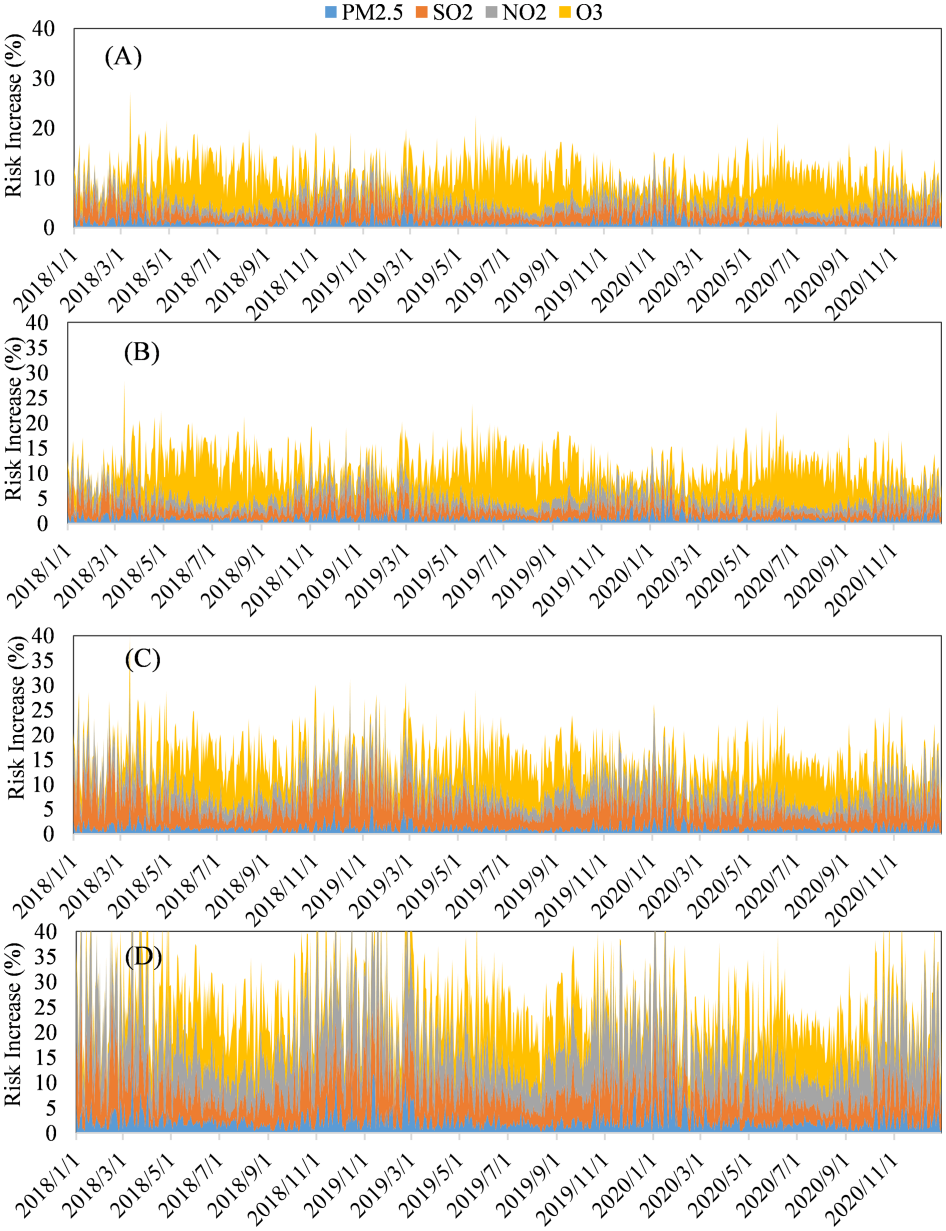
Contributions of each air pollutant to increased daily mortality among different populations in Tianjin 2018–2020: **(A)** Total population. Populations with **(B)** cardiovascular and cerebrovascular diseases **(CD)**, **(C)** lung cancer (LC), and **(D)** chronic respiratory diseases (CRD).

Figure 7 shows the contributions of each air pollutant to increased daily mortality among different populations in Tianjin 2018–2020. According to the figure, for the total population, the total contributions of each air pollutant to increased daily mortality were 4.19–27.21%. For the populations with cardiovascular and cerebrovascular diseases (CD), lung cancer (LC), and chronic respiratory diseases (CRD), the total contributions to increased daily mortality were 4.28–28.24%, 5.94–39.73%, and 9.25–71.10%. The effects of each air pollutant on the health of populations with chronic respiratory disease, lung cancer and cardiovascular disease were more significant than those of the whole population. During the summer (June to August), the contribution of O_3_ (the yellow part in the Figure 7) to the daily mortality rate of different populations significantly increased. The health effects of increased O_3_ concentrations during the summer were significantly higher. SO_2_ contributed the highest proportion to the increase in daily mortality rate of lung cancer populations, ranging from 6.86 to 69.24%. And the proportion of SO_2_ contributions to the increase in daily mortality rates of the total populations, cardiovascular and cerebrovascular diseases populations, and respiratory diseases populations were 4.00–59.85%, 3.24–54.69%, and 6.03–59.62%. Compared with other populations, NO_2_ and PM_2.5_ had greater contributions to the increase in daily mortality rate in chronic respiratory diseases populations, with an average of 9.47 and 2.43%, respectively.

#### 3.4.2. Construction of AQHI index for populations with different diseases in Tianjin

The exposure-response relationships among populations with different diseases clarified that populations with lung cancer, cardiovascular and chronic respiratory disease are more sensitive to the acute health effects of air pollution.

**Figure 8:**
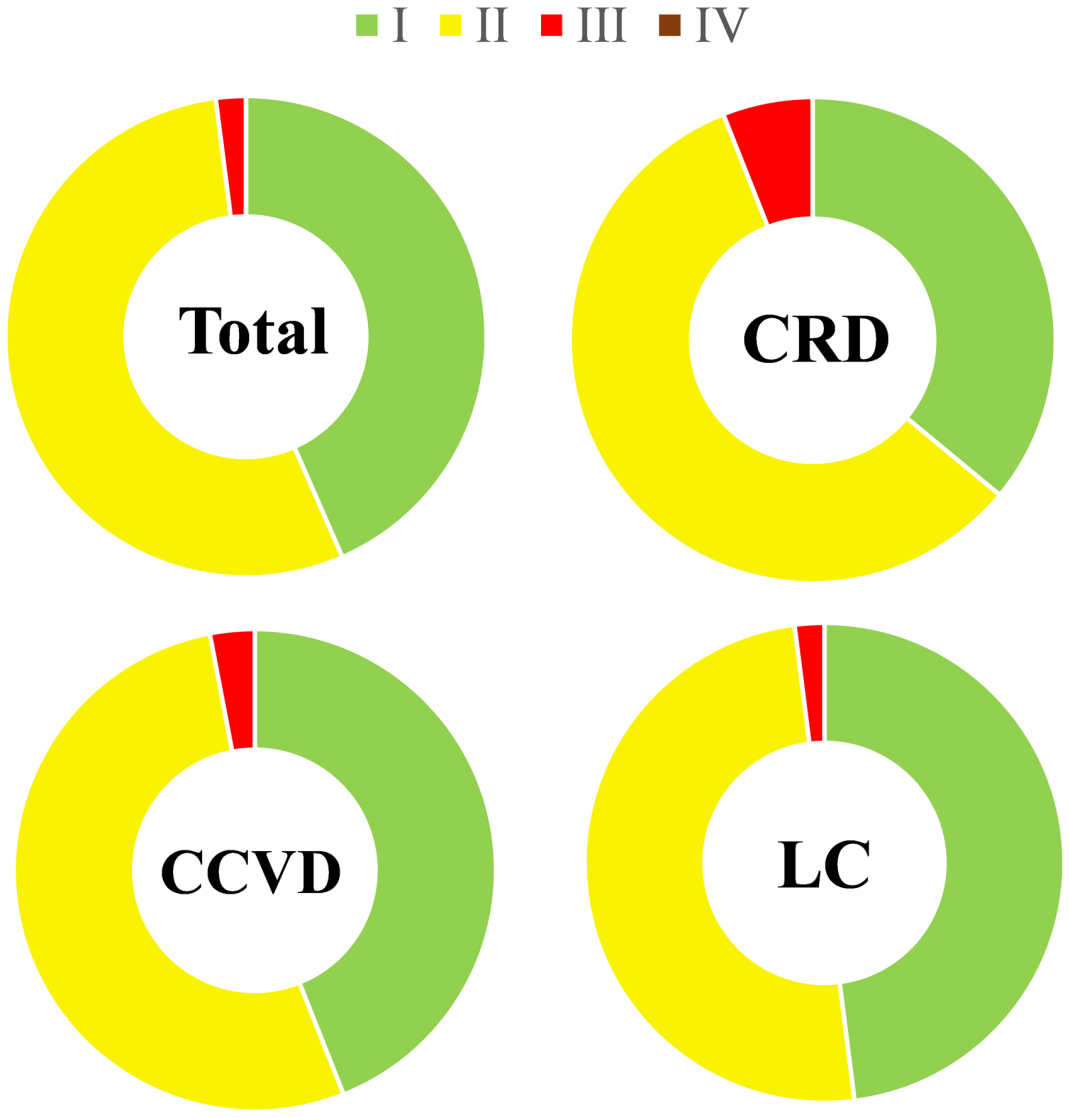
Comparative AQHI and S-AQHI grades for health risk in Tianjin 2018–2020. Levels I, II, III and IV are low, moderate, high, and severe, respectively.

The resulting AQHI values of air pollutants in Tianjin for populations with different types of diseases were graded according to the criteria shown in Table 1. Figure 8 shows that the grades of the AQHI and S-AQHI indices of Tianjin for 2018–2020 varied, and that the number of days with moderate and high grades were worse for populations with cardiovascular and chronic respiratory diseases than for the whole population, being 53, 3, and 58%, and 6%, respectively.

## 4. Discussion

### 4.1. Health risk evaluation of air pollutants in Tianjin

The health effects results of air pollutants indicated that air pollution in Tianjin is influenced by meteorological factors and that the level of pollution remains very high, and the seasonal variations in PM_2.5_, SO_2_, NO_2_, O_3_, and CO are clear, whereas the trends of AQHI and CRI-AQHI are not the same as those of the air pollutant concentrations, because the AQHI takes into account the combined health effects of multiple air pollutants, resulting in less distinct seasonal variations.

The validity analysis results indicated closer correlations between the exposure-response relationship of total mortality and the AQHI and the CRI-AQHI compared with the AQI. And according to the analysis of excess mortality rate, the correlations between AQHI and CRI-AQHI and health are significantly stronger than AQI, indicated that AQHI and CRI-AQHI are more effective in predicting daily mortality rates of residents. It is similar to the previous research results. Du etc. (22) found that the associations of AQHI with mortality were stronger than AQI. Cao et al. (23, 37) also found that CRI-AQHI and AQHI performed better than AQI when identifying health risks.

### 4.2. Health effects analysis of different disease groups

The acute health effects of air pollution are influenced by several factors, and public health prevention can be implemented for populations with various types of diseases to minimize the health hazards of air pollution. This study showed that people with lung cancer, cardiovascular and chronic respiratory diseases are more susceptible to air pollution, and that chronic underlying cardiopulmonary and respiratory diseases enhance sensitivity to air pollution. In fact, individuals with diseases have inherently poor health with diminished ability to clear inhaled pollutants from the respiratory tract and decreased immunity, and are thus more susceptible to pollutants (28, 29).

The health effects of each air pollutant on the population with chronic respiratory disease was the most significant, followed by lung cancer and cardiovascular disease. The contribution of each air pollutant to the increase in daily mortality rates was similar between the entire population and the population with cardiovascular disease. The health effects of increased O_3_ concentrations during the summer were significantly higher. The contribution of SO_2_ to the increase in daily mortality rate for the population with lung cancer was larger than that for other populations, and SO_2_, as an auxiliary carcinogen, is mainly generated by pollution due to coal combustion which is more severe when air convection is very weak or during an atmospheric inversion. Epidemiological studies have shown that SO_2_ can affect lung function and is closely associated with lung cancer mortality (38–40). Sulfur dioxide can adsorb onto the surface of PM_2.5_ and deeply penetrate the respiratory tract, increasing the level of toxicity 3–4-fold, and it can be oxidized to SO_3_ through catalysis by metal particles (39). This is 4-fold more dangerous than SO_2_ alone. Furthermore, SO_2_ combined with benzo(a)pyrene can increase the carcinogenicity of the latter. Both NO_2_ and PM_2.5_ contribute more to the increase of daily mortality rates of persons with chronic respiratory disease than other populations. Having entered the lungs, PM_2.5_ blocks local tissues, causing a decrease in local bronchial ventilation and a loss of fine bronchial and alveolar air exchange. Adsorbed with harmful gasses, PM_2.5_ can irritate or corrode the alveolar wall, and the long-term effect can damage the respiratory defense function, leading to bronchitis, emphysema and bronchial asthma. Epidemiological findings have shown that short- and long-term exposure to high concentrations of particulate matter can increase the morbidity and mortality rates of respiratory diseases (41, 42). Nitrogen dioxide is mainly produced during the combustion of nitrogenous substances, and the main source is vehicle exhaust emissions. Inhalation of NO_2_ not only damages human lung function, but also causes bronchial asthma and other diseases (43, 44). Epidemiological findings have associated NO_2_ with increased rates of mortality from chronic respiratory diseases (43, 44). In addition, several cohort study also shows that long-term exposure to PM_2.5_ could significantly increase the incidence rate of total cardiovascular diseases among residents (HR = 1.04, 1.02–1.07) (45), and long-term exposure to PM_2.5_ and nitrogen dioxide have an impact on multiple stages of the development of cardiovascular metabolic comorbidity (46). Long term exposure to ozone is associated with an increased risk of total cardiovascular disease death, ischemic heart disease death, and stroke death. For every 10 μg/m^3^ increase in ozone concentration during the warm season, these death risks increase by 9.3, 18.4, and 6.3%, respectively (47). This shows that air pollutants are harmful to humans in many ways, and the impact of each on human health cannot be taken lightly.

The above results show that the impact of each air pollutant on the health of populations with different types of diseases varies. Therefore, it is necessary to establish S-AQHI for these populations in this study. The results also provide technical support for the feasibility and effectiveness of establishing S-AQHI for other susceptible populations (pregnant women, children, older adults group, etc.). S-AQHI can be established for various susceptible populations to comprehensively assess public health risks in the future.

### 4.3. Strengths and limitations

In this study, we constructed Tianjin AQHI and a validity comparison based on two different methods, and constructed S-AQHIs for populations with different disease. In addition, we analyzed the health effects of air pollution based on different disease groups. The present results will provide guidance for the general and diseases populations to adopt of healthy behaviors, and will serve as a model for the establishment of AQHI in other Chinese cities in the future.

The AQHI is an indicator of the combined health effects of multiple air pollutants. Thus, the selection of appropriate pollutant data, health data and modeling methods as primary indicators is critical for establishing an AQHI for different regions and populations, but the types of included pollutants and modeling methods have not yet reached consensus. For example, To etc. (29) developed an AQHI for chronic diseases in Canada based on exposure-response relationships between each of PM_2.5_, NO_2_, and O_3_ and the number of patients with various diseases. Li etc. (15) developed an AQHI for Guangzhou using a single-pollutant model based on exposure-response relationships between daily mortality rates and each of PM_2.5_, O_3_, NO_2_, and SO_2_. Zeng etc. (20) created an AQHI for Tianjin using principal component analysis (PCA), comprehensively considering the exposure-response relationship between each of PM_2.5_, PM_10_, SO_2_, NO_2_, CO, and O_3_ and daily mortality as well as years of life lost. Pollutant screening, modeling methods, and the selection of health data used to establish an AQHI in various regions clearly differ. This has also led to a lack of comparability among studies associated with AQHI. Therefore, relevant ambient air quality standards and policies for AQHI should be established to comprehensively analyze the combined health effects of air pollutants in different regions. Residents in all regions should be educated about air pollution and the severity of its potential harmful effects on health.

Due to the data acquisition limitations, this study used disease mortality data for different disease groups, which were not comprehensive enough in analyzing health risks. In the future, different disease incidence data can be obtained to assess health risks more comprehensively and effectively.

## 5. Conclusion

We constructed an AQHI and a CRI-AQHI for Tianjin using single- and multi-pollutant models and environmental (PM_2.5_, O_3_, NO_2_, SO_2_), meteorological, and health data for 2018–2020. Compared with the AQI, the AQHI and CRI-AQHI established herein correlated more closely with exposure-response relationships that were mostly linear. Each increase in the IQRs of AQHI, CRI-AQHI and AQI values resulted in an increase of 2.06, 1.69 and 0.62%, respectively, in total daily mortality rates. These findings confirmed that the AQHI and CRI-AQHI were far more effective than AQI in predicting the daily mortality rates of Tianjin residents and that they were similarly associated with health. Therefore, we selected the coefficients of the single-pollutant model when establishing the AQHI for Tianjin to maintain comparability with previous AQHI findings.

Based on these findings, we used our AQHI to analyze populations with different types of diseases in Tianjin. Populations with lung cancer, cardiovascular and chronic respiratory disease were vulnerable, and more susceptible to air pollution. The deleterious effects of each air pollutant on the health of the population with chronic respiratory disease were the most significant, followed by lung cancer and cardiovascular and cerebrovascular diseases. The contributions of each air pollutant to the increased daily mortality rates of the entire population and the population with cardiovascular and cerebrovascular diseases were similar, with O_3_ having significantly worse effects on health during the summer. Sulfur dioxide contributed more to the increase in daily mortality rates of the population with lung cancer than other types of diseases. Nitrogen dioxide and PM_2.5_ contributed more to the increase in daily mortality rates for populations with chronic respiratory than other diseases. Based on these findings, we established an S-AQHI for various populations with different diseases. The present results will provide guidance for the general and diseases populations to adopt of healthy behaviors. In the future, the combined health effects of air pollutants in various regions will be comprehensively analyzed. Relevant ambient air quality standards and policies for AQHI need to be established to comprehensively analyze the combined health effects of air pollutants in various regions, and to educate the public in general to recognize and understand the severity of air pollution and its potential effects on health.

## Data availability statement

The raw data supporting the conclusions of this article will be made available by the authors, without undue reservation.

## Author contributions

YW, DD, and MS contributed to conception, design of the study, and wrote the first draft of the manuscript. YW organized the database. YW and DD performed the statistical analysis. MD, YD, LG, and ZX wrote sections of the manuscript. All authors contributed to manuscript revision, read, and approved the submitted version.

## Funding

This work was supported by China’s National Key Research and Development Program (2019YFE0194500) and the Municipal Financial Research Project of Beijing Academy of Science and Technology (11000022T000000445296 and 11000022T000000468149).

## Conflict of interest

The authors declare that the research was conducted in the absence of any commercial or financial relationships that could be construed as a potential conflict of interest.

## Publisher’s note

All claims expressed in this article are solely those of the authors and do not necessarily represent those of their affiliated organizations, or those of the publisher, the editors and the reviewers. Any product that may be evaluated in this article, or claim that may be made by its manufacturer, is not guaranteed or endorsed by the publisher.

## Footnotes

^1^http://www.tianqihoubao.com/aqi/
