# Supplementary material for: Evaluation of the health risk using multi-pollutant air quality health index: case study in Tianjin, China
Source: Front Public Health. 2023 Jun 7;11:1177290. doi: 10.3389/fpubh.2023.1177290 (PMC10289283; doi:10.3389/fpubh.2023.1177290)
Supplement: Supplementary file 1 [file Data_Sheet_1.docx]

Supplementary Material

Evaluation of the health risk using multi-pollutant air quality health index: Case study in Tianjin, China

Yu Wang^1,2^ , Mo Dan^2^, Yan Dou^2^, Ling Guo^2^, Zhizhen Xu^2^, Ding Ding^2,3*^, Mushui Shu^2*^

^1^Center of Excellence for Environmental Safety and Biological Effects, Beijing Key Laboratory for Green Catalysis and Separation, Department of Chemistry, Beijing University of Technology, Beijing 100124, P. R. China.

^2^Institute of Urban Safety and Environmental Science, Beijing Academy of Science and Technology, Beijing 100054, China.

^3^School of Energy and Environmental Engineering, University of Science and Technology Beijing, Beijing 100083, China.

*** Correspondence:**Corresponding Author
Ding Ding^*^ [d202110092@xs.ustb.edu.cn](mailto:d202110092@xs.ustb.edu.cn)

Mushui Shu^*^ [sms@bmilp.com](mailto:sms@bmilp.com)

# Supplementary Figures and Tables

**Table S1.** Summary of environmental variables and daily non-accidental deaths in Tianjin from 2018 to 2020

| Variables | Mean | SD | Min | P25 | Median | P75 | Max | IQR |
| --- | --- | --- | --- | --- | --- | --- | --- | --- |
| PM_2.5_(μg/m^3^) | 50.31 | 38.7 | 5.5 | 25.4 | 40.2 | 61.3 | 276.4 | 35.9 |
| PM_10_(μg/m^3^) | 78.74 | 47.34 | 9.1 | 47 | 66.5 | 96.9 | 410 | 49.9 |
| SO_2_(μg/m^3^) | 10.13 | 5.49 | 2.1 | 6.1 | 8.8 | 12.9 | 37.5 | 6.8 |
| CO(mg/m^3^) | 0.96 | 0.42 | 0.3 | 0.7 | 0.9 | 1.1 | 3.1 | 0.4 |
| NO_2_(μg/m^3^) | 41.95 | 18.96 | 8.7 | 27.8 | 37.1 | 53.2 | 134.1 | 25.4 |
| O_3_(μg/m^3^) | 123.74 | 67.46 | 4.3 | 67.9 | 107 | 170.7 | 350.1 | 102.8 |
| AVET(℃) | 15.21 | 10.92 | -10 | 5 | 16 | 26 | 33 | 21 |
| AVEH(%) | 52.19 | 18.49 | 12 | 38 | 53 | 66 | 98 | 28 |
| Mortality | 206 | 29 | 142 | 185 | 202 | 222 | 308 | 37 |


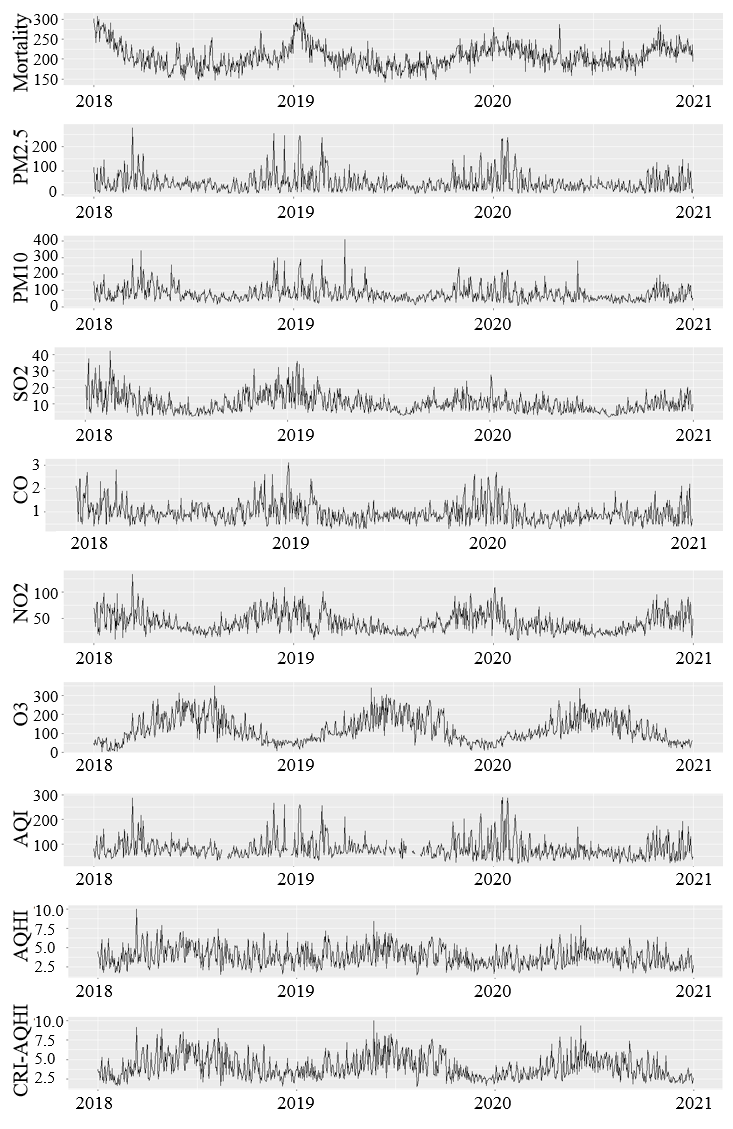


**Figure S1.** Daily trends of air pollutants, mortality, the AQI, and the AQHI in Tianjin from 2018 to 2020.
